# Identification of Differentially Expressed Proteins in Murine Embryonic and Postnatal Cortical Neural Progenitors

**DOI:** 10.1371/journal.pone.0009121

**Published:** 2010-02-09

**Authors:** Lorelei D. Shoemaker, Nicholas M. Orozco, Daniel H. Geschwind, Julian P. Whitelegge, Kym F. Faull, Harley I. Kornblum

**Affiliations:** 1 Pasarow Mass Spectrometry Laboratory, David Geffen School of Medicine, University of California Los Angeles, Los Angeles, California, United States of America; 2 Neuropsychiatric Institute - Semel Institute for Neuroscience & Human Behavior and Department of Psychiatry and Biobehavioral Sciences, David Geffen School of Medicine, University of California Los Angeles, Los Angeles, California, United States of America; 3 Department of Neurology, David Geffen School of Medicine, University of California Los Angeles, Los Angeles, California, United States of America; 4 Department of Molecular and Medical Pharmacology, David Geffen School of Medicine, University of California Los Angeles, Los Angeles, California, United States of America; 5 Brain Research Institute, David Geffen School of Medicine, University of California Los Angeles, Los Angeles, California, United States of America; University of Nebraska, United States of America

## Abstract

**Background:**

The central nervous system (CNS) develops from a heterogeneous pool of neural stem and progenitor cells (NSPC), the underlying differences among which are poorly understood. The study of NSPC would be greatly facilitated by the identification of additional proteins that mediate their function and that would distinguish amongst different progenitor populations.

**Methodology/Principal Findings:**

To identify membrane and membrane-associated proteins expressed by NSPC, we used a proteomics approach to profile NSPC cultured as neurospheres (NS) isolated from the murine cortex during a period of neurogenesis (embryonic day 11.5, E11.5), as compared to NSPC isolated at a peak of gliogenesis (postnatal day 1, P0) and to differentiated E11.5 NS. 54 proteins were identified with high expression in E11.5 NS, including the TrkC receptor, several heterotrimeric G proteins, and the Neogenin receptor. 24 proteins were identified with similar expression in E11.5 and P0 NS over differentiated E11.5 NS, and 13 proteins were identified with high expression specifically in P0 NS compared to E11.5 NS. To illustrate the potential relevance of these identified proteins to neural stem cell biology, the function of Neogenin was further studied. Using Fluorescence Activated Cell Sorting (FACS) analysis, expression of Neogenin was associated with a self-renewing population present in both E11.5 and adult subventricular zone (SVZ) NS but not in P0 NS. E11.5 NS expressed a putative Neogenin ligand, RGMa, and underwent apoptosis when exposed to a ligand-blocking antibody.

**Conclusions/Significance:**

There are fundamental differences between the continuously self-renewing and more limited progenitors of the developing cortex. We identified a subset of differentially expressed proteins that serve not only as a set of functionally important proteins, but as a useful set of markers for the subsequent analysis of NSPC. Neogenin is associated with the continuously self-renewing and neurogenic cells present in E11.5 cortical and adult SVZ NS, and the Neogenin/RGMa receptor/ligand pair may regulate cell survival during development.

## Introduction

The central nervous system (CNS) develops from a population of neural stem and progenitor cells (NSPC) in a spatially and temporally defined manner, with prenatal neurogenesis followed by a wave of postnatal gliogenesis, to generate the appropriate architecture, and types and number of cells of which the mature CNS is compromised [1], [2]. As cortical development proceeds, NSPC shift from being highly proliferative and self-renewing to being relatively quiescent, reducing their overall number either through a series of non-renewing symmetrical cell divisions, developmental programmed cell death, or perhaps even migration [3], [4], [5]. Mounting evidence suggests that NSPC isolated from spatially and temporally distinct regions are fundamentally different in terms of self-renewal capacity, potential and propensity to generate certain cell types [6], [7], [8], [9]; however, the study of these populations of NSPC is hampered by the limited number of identified molecules that define these subpopulations of cells.

Gene expression analysis has identified transcriptional differences that exist amongst various populations of NSPC and several candidate stem and progenitor genes have been identified [10], [11], [12], [13], [14]. Proteomics approaches have the advantage of examining expression differences that may not be under transcriptional control [15], [16], and several studies have been undertaken to profile neural stem cell protein expression, including analysis of a neural stem cell line [17], differentiating adult hippocampal and subventricular zone (SVZ) neural stem cells [18], [19], [20], differentiating porcine neural stem cells [21], and a comparison of adult SVZ and olfactory bulb progenitors [22].

To identify proteins that may define subpopulations of NSPC, we chose to compare membrane and membrane-associated protein expression profiles of cortical neurospheres (NS) generated during a highly neurogenic period (embryonic day 11.5, E11.5) and during a gliogenic period (postnatal day 1, P0). The potential and longevity of these NS cultures was characterized, with E11.5 NS reflective of a more stem cell-like population, and the P0 NS, of a more restricted progenitor. Using protein expression analysis, we identified differences in membrane and membrane-associated proteins expressed by these populations of NS, including the receptor, Neogenin, which may have different functions as development proceeds and which may be a marker for an early embryonic cortical NSPC. These experiments demonstrate fundamental differences between embryonic and postnatal cortical NSPC, and provides a list of candidate membrane and membrane-associated proteins expressed by NSPC.

## Results

### E11.5 Cortical NS Contain Persistently Self-Renewing, Neurogenic NSPCs while P0 Cortical NS Contain Progenitors with a Limited Capacity for Self-Renewal and Neurogenesis

**Figure 1:**
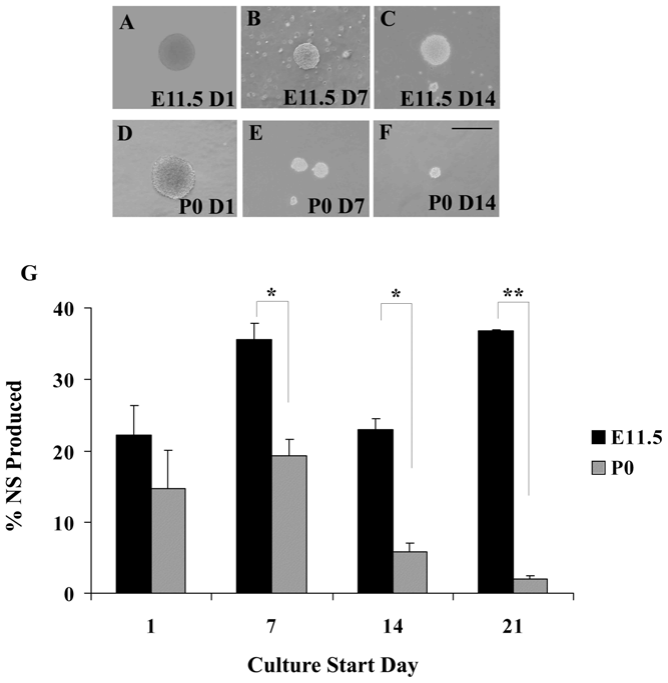
E11.5 NS cultures are highly proliferative while P0 cultures show reduced proliferation. Representative phase contrast images of NS generated from E11.5 (A–C) and from P0 (D–F) cultures, started on D1 (A, D), D7 (B, E) and on D14 (C, F). Scale bar, 50 µm. G.) NS produced as a percentage of the original plating density from E11.5 and P0 cultures as a function of culture start day. The data represent n = 5 independent experiments. Results are presented as the mean % NS produced +/− standard error of the mean (SEM). * p≤0.05 * * p≤0.01

To validate the cell source for the subsequent proteomics experiments, NS cultures from E11.5 and P0 cortex were characterized according to proliferation, multipotentiality and longevity in culture. To examine the proliferation of E11.5 and P0 NS cultures with time, low density cultures (1,000 cells/ml) were generated from three separate *in vitro* time points: acutely isolated tissue, and following 7 d, 14 d, and 21 d of growth at high density (50,000 cells/ml), as outlined in Figure S1. While the NS derived from E11.5 and from P0 were similar in number, size and overall appearance at D1 (Figure 1A, D), by D14, P0 NS were considerably smaller and fewer in number (Figure 1C, F). The ability of P0 cultures to generate NS diminished with time, from 15% at D1 to 2% by D21. Low density E11.5 cultures (Figure 1G) continued to produce similar percentages of NS however, ranging from 22% at D1 to 37% at D21. These differences between E11.5 and P0 were significant by D7 and continued to be significant to D21. The slight reduction in NS production at D14 for E11.5 NS (which is not statistically significant), is likely a function of *in vitro* variability, as the E11.5 NS cultures continue to proliferate well beyond D21 (data not shown).

**Figure 2:**
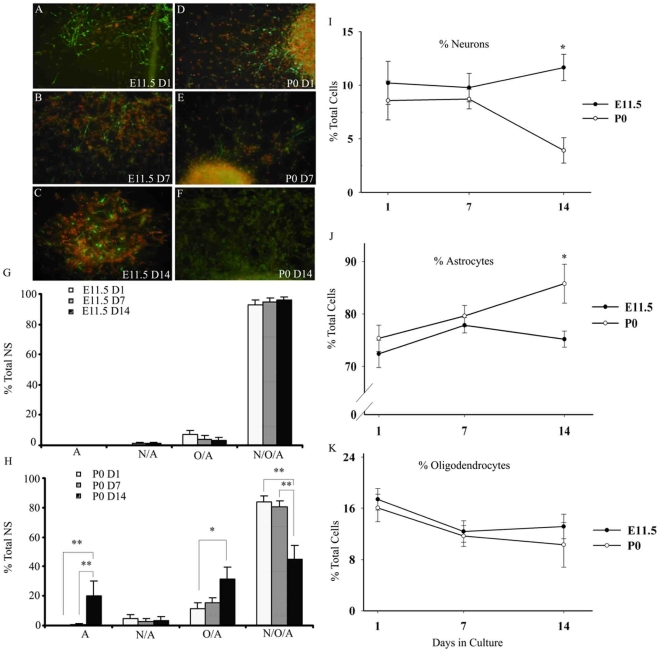
Potency and production of the three main neural cell lineages from E11.5 and P0 NS upon differentiation. Representative fluorescent images of neurons from differentiated E11.5 NS are shown in A–C and P0 NS in D–F (neurons in green in all panels, nuclei in red in panels B, C, D, E, and astrocytes in red in panels A and C). E11.5 (G) and P0 (H) individual differentiated NS derived from D1, D7 and D14 cultures were scored for presence of Tuj1-, O4-, and GFAP-immunoreactive cells: ‘A’, astrocytes only; ‘N/A’ - both neurons and astrocytes; ‘O/A’ – both oligodendrocytes and astrocytes; ‘N/O/A’ – presence of all three cell types. Significant differences in the generation of di- and multi-potent NS existed between E11.5 (G) and P0 (H) NS cultures, including an increase in P0 NS containing only O/A at Day 7 (p<0.05), and decreases in tripotent P0 NS at Day 7 (p<0.05) and at Day 14 (p<0.01). Results are presented as the % of total NS +/− SEM. The percent of neurons (I), astrocytes (J), and oligodendrocytes (K) produced by E11.5 (shaded symbols) and P0 NS (open symbols) as a function of time in culture was evaluated by scoring random fields. Results are presented as the mean % of total cells +/− SEM. Note different y-axis scale for I, J, and K. * p≤0.05 * * p≤0.01

To determine if there were also differences in the ability to generate the three main cell types of the CNS, differentiated NS were scored for immunoreactivity for markers of neurons (Tuj1), oligodendrocytes (O4) and astrocytes (GFAP). E11.5 cultures from D1, D7 and D14 predominately and consistently produced tripotent NS containing all three major cell types of the CNS upon differentiation (Figure 2G), ranging from 93% at D1 to 96% by D14, with a small percentage of NS containing only oligodendrocytes and astrocytes or neurons and astrocytes. However, P0 cultures lost the ability to generate tripotent NS, with an approximately 50% reduction from D1 to D14 (Figure 2H) and a subsequent significant increase in the production of NS containing cells of the glial lineage: oligodendrocytes and astrocytes (from 12% to 31%), or astrocytes alone (from 0% to 20%). Production of neuron- and astrocyte-containing P0 NS remained below 4%. Significant differences in the generation of di- and multipotent NS existed between E11.5 and P0 NS cultures, including an increase in P0 NS containing only oligodendrocytes and astrocytes at Day 7 (p<0.05), and decreases in tripotent P0 NS at Day 7 (p<0.05) and at Day 14 (p<0.01).

To further reveal differences in the neurogenic and gliogenic capacity of the embryonic and postnatal cultures, the percent of each cell type produced upon differentiation was determined. Low density E11.5 D1, D7 and D14 cultures produced all three neural cell lineages, with no significant differences in the percent of cell type produced over time (Figure 2I, J, K). In P0 cultures, however, there was a significant decrease in the production of neurons from 9% on D1 to 4% by D14 (Figure 2I), and a concomitant increase in the generation of astrocytes, from 75% at D1 to 86% by D14 (Figure 2J). The production of oligodendrocytes from both cultures ranged from 17% to 10% of the total cells and despite this apparent decreasing trend, the data did not reach statistical significance (Figure 2K). Representative fluorescence images of neurons generated in these cultures are in Figure 2A–F. Thus, taken in sum, our data indicate that E11.5 NS contain a self-renewing population of multipotent stem-like cells, while the P0 NS contains progenitors with diminished self-renewal and neurogenic capacity.

### Protein Expression Profiles by 2DGE Reveal Differentially Expressed Proteins between Embryonic and Postnatal NS

**Figure 3:**
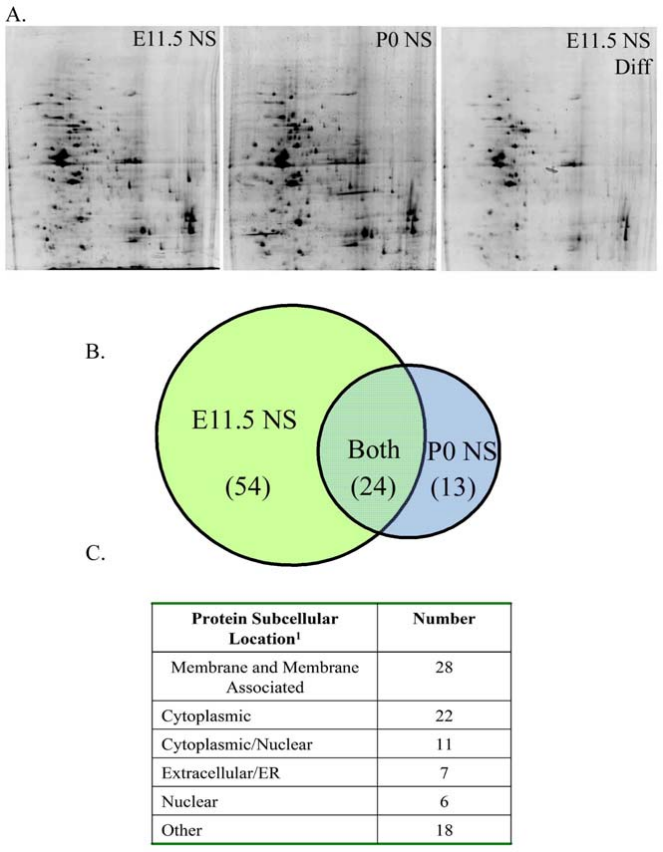
Summary of differential protein expression by 2D gel electrophoresis in a membrane-enriched preparation of E11.5 and P0 NS. SyproRuby-stained representative 2D gels (A) of membrane-enriched fractions from E11.5 NS, P0 NS, and E11.5 differentiated NS. The Venn diagram (B) summarizes the number of identified proteins with differential expression between E11.5 NS and P0 NS. Overlap between the two sets indicates those proteins expressed by both E11.5 and P0, yet increased over E11.5 differentiated NS. The predicted sub-cellular locations of these proteins according to ^1^ WoLF PSORT (wolfpsort.org) are listed in (C).

To identify proteins that define neurogenic E11.5 NS, membrane enriched fractions from three groups of cells were compared by 2DGE (Figure 3A): E11.5 NS, E11.5 differentiated NS, and P0 NS. A membrane-enriched sub-cellular fraction was chosen to enable detection of proteins that, upon further examination, may prove useful as stem/progenitor cell markers. The first of two comparisons was between undifferentiated and differentiated E11.5 NS cultures, to yield those proteins expressed by proliferative, neurogenic cells, over those proteins more highly expressed in differentiated, postmitotic cells. The second comparison was between undifferentiated E11.5 NS and P0 NS to identify those proteins specifically expressed in a neurogenic population of cells over a more restricted population (P0 NS), thereby taking into account those proteins related strictly to proliferation. Protein spots with a greater than 2-fold difference in intensity were excised, digested and the proteins identified using μLC-MS/MS. This workflow is illustrated in Figure S2 and a typical ion chromatogram and MS/MS spectrum are illustrated in Figure S3. While not all differentially expressed proteins were identified (primarily as a result of poor quality MS/MS data and/or low confidence MS/MS scores), 54 proteins were identified with high expression in the proliferative, neurogenic E11.5 NS over both differentiated E11.5 NS and the restricted P0 NS, 24 proteins were comparably expressed in E11.5 and P0 NS over differentiated E11.5 NS, and 13 proteins were highly expressed specifically in P0 NS compared to E11.5 NS (Figure 3B). A partial list of those proteins highly expressed in E11.5 NS and in P0 NS can be found in Table 1. An additional set of proteins were highly expressed in both E11.5 and P0 NS populations over differentiated E11.5 NS cultures and may reflect common functions in proliferating cells (Table S1). A fully annotated list of all proteins, including identified peptides and bioinformatic identification scores, can be found in Table S2. As Figure 3C illustrates, of the 91 proteins identified, approximately 31% were membrane or membrane-associated proteins. To verify the expression pattern of proteins observed in 2DGE, expression levels for the proteins TrkC, RACK1 and HSP90, were analyzed by Western blot analysis (Figure S4).

**Table 1:** Partial list of proteins with higher expression in E11.5 NS compared to P0 NS, as well as those proteins with higher expression in P0 NS as compared to E11.5 NS. Proteins were identified by μLC-MS/MS. Detailed information regarding protein identification can be found in Table S2.

Protein Name	Gene Symbol	UniProtKB/TrEMBL Number
**Higher Expression In E11.5 NS**		
Voltage-dependent anion channel 2	Vdac2	Q99L98
Neogenin	Neo1	P97798
Seizure related gene 6	Sez6	Q7TSK2
Sodium-calcium exchanger	Slc8a1	O35157
Neurotrophic tyrosine kinase receptor	TrkC	Q6VNS1
TGF-beta receptor type III	Tgfbr3	O88393
Guanine nucleotide-binding protein	Gnao1	P18872
Guanine nucleotide-binding protein	Gna12/Gna13	P27600
GTP-binding protein REM 1	Rem1	O35929
Guanine nucleotide binding protein	Gnb2-rs1	Q5NCC6
Guanine nucleotide-binding protein	Gnb2l1	Q9CSQ0
Similar to interleukin 17 receptor E	Il17re	Q6AZ51
Hypothetical protein C6B12.02c	SPAC6B12.02c	O14207
Receptor tyrosine-protein kinase	Erbb2	Q6ZPE0
Alpha 3 catenin	Ctnna3	Q8C0N3
FK506 binding protein 9	Fkbp9	Q80ZZ6
**Higher Expression In P0 NS**		
Cyclic nucleotide gated channel alpha 2	Cnga2	Q80XH6
Selectin P	SELP	Q5TI45
ATPase, H+ transporting	Atp6v0d1	Q921S5
Arsenical pump-driving ATPase	Asna1	O54984
(smad8/smad9) Mothers against decapentaplegic homolog 9	Smad9	Q9JIW5
Down-regulated by Ctnnb1, a	Drctnnb1a	Q6P9N1
Tumor rejection antigen gp96	Tra1	Q8CCY5
Early endosome antigen 1	Eea1	Q8BL66

### Neogenin, a Single Transmembrane Receptor, Is Highly Expressed in E11.5 NS but Is Not Transcriptionally Regulated

**Figure 4:**
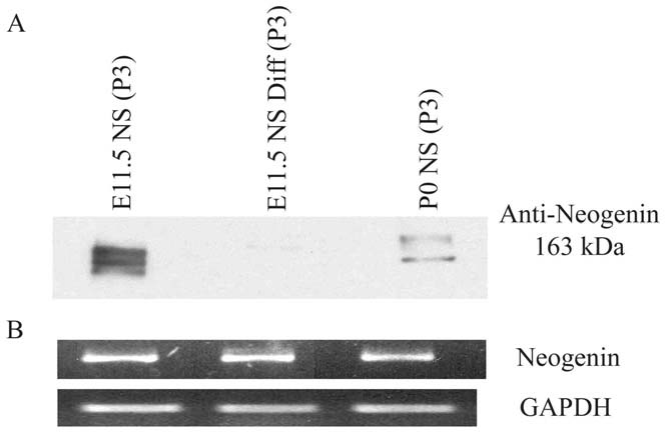
Neogenin is highly expressed in E11.5 NS. Neogenin protein was expressed at high levels in the membrane enriched fraction of E11.5 NS over P0 NS, and decreased upon differentiation (A), while mRNA levels appeared to be expressed at equal levels (B). Equal amounts of protein and of RNA were loaded into each well, with GAPDH used as a control for the semi-quantitative PCR.

Neogenin was highly expressed in E11.5 NS, as confirmed by western blot analysis of membrane-enriched fractions from E11.5 NS, E11.5 Differentiated NS and P0 NS (Figure 4A). Semi-quantitative PCR demonstrates that these differences in expression are not reflected by differences in transcript level (Figure 4B), despite one report of transcript expression differences during development [23].

To characterize the Neogenin-expressing cells, E11.5 and P0 NS were examined by immunocytochemistry for co-expression of Nestin, a putative marker for neural stem and progenitor cells, and Neogenin (Figure S5A, B). Neogenin was expressed in 54% of the total cells in dissociated E11.5 NS, and by 43% of dissociated P0 NS (Figure S5B). However, in E11.5 NS, Neogenin and Nestin co-expression occurred in 40% of the cells, while co-expression occurred in only 26% of the cells in P0 (Figure S5B). These data suggest that Neogenin expression is more highly associated with progenitors in the E11.5-derived NS.

### Neogenin Expression Is Associated with Long-Term NS-Forming Cells in E11.5 and Adult SVZ NS

**Figure 5:**
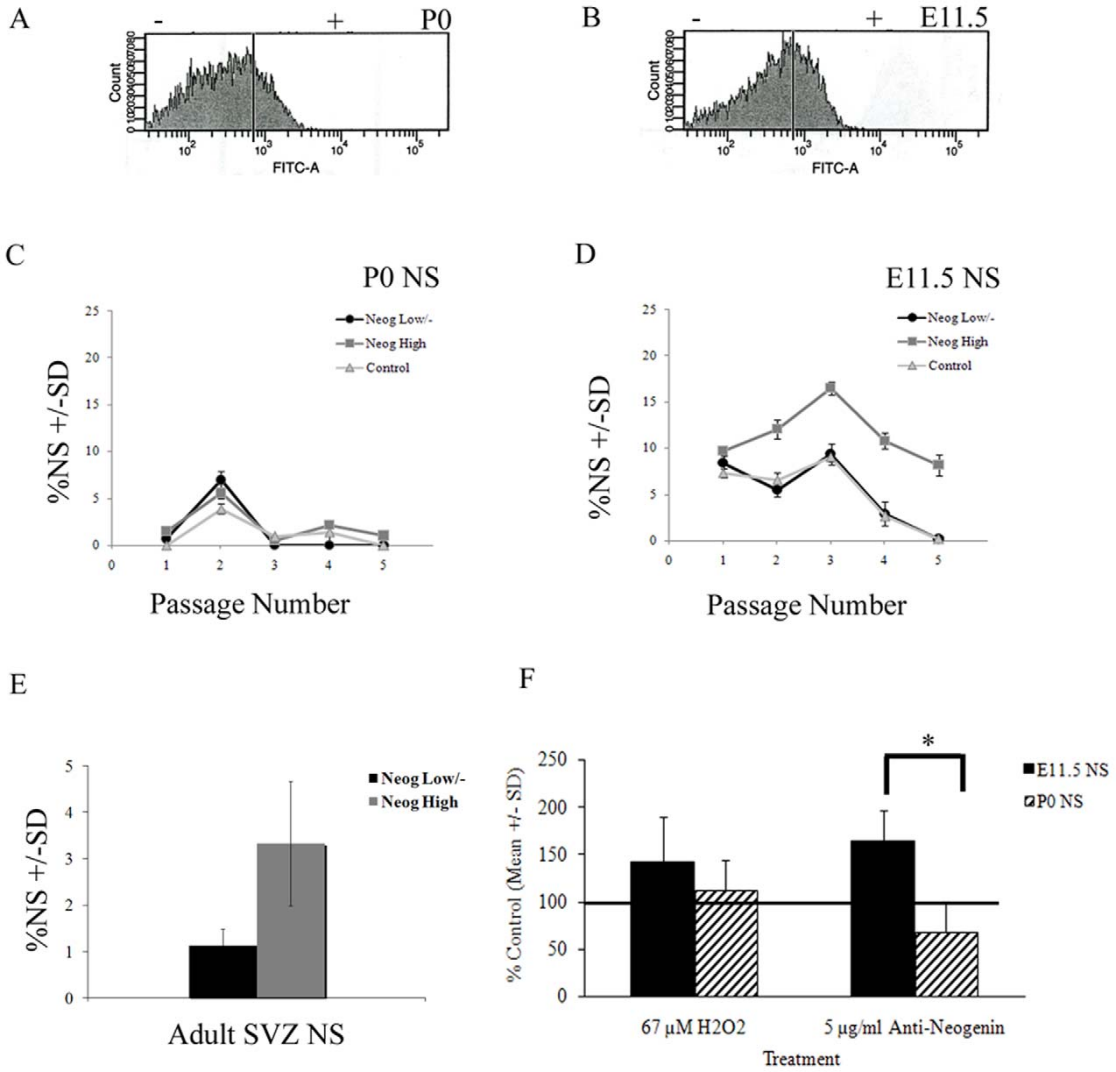
Neogenin expression is associated with a highly proliferative cell population in E11.5 NS, while receptor-ligand blocking results in cell death. Mature secondary NS from both P0 (B) and E11.5 (A) cortex were dissociated and sorted according to levels of Neogenin expression. Cells were grown at 5,000 cells/ml (Passage 1–2 in C, D), followed by passages at 1,000 cells/ml (Passage 3–5 in C, D). FACS experiments were replicated with a second culture with similar results. Mature tertiary adult SVZ NS were sorted according to levels of Neogenin expression, and cultured for 14 d (E). Experiments were performed in triplicate. Results are presented as the % NS generated +/− standard deviation. Addition of ligand-blocking antibody resulted in increased activated Caspase3/7 in E11.5 NS (F). Anti-Neogenin antibody was added at a concentration of 5 µg/ml and cells were exposed to antibody for 24 h. Results are presented as the percent of control activated Caspase3/7, using hydrogen peroxide as a positive control treatment, and PBS and mouse IgG as negative control treatments. *p≤0.05.

Using an antibody to the extracellular portion of Neogenin that does not block ligand binding [24] and fluorescence activated cell sorting (FACS), dissociated E11.5, P0 and adult SVZ NS were sorted according to their Neogenin expression, resulting in a Neogenin-high and a Neogenin-low/negative population (Figure 5A, B). To determine if Neogenin expression is associated with longevity in culture, these sorted populations were grown in culture and assessed for ability to generate NS. While there was no growth advantage associated with Neogenin expression for P0 cells (Figure 5C), E11.5 (Figure 5D) and adult SVZ (Figure 5E) Neogenin-high cells consistently produced a higher percentage of NS than the Neogenin-low and unsorted cells. NS production shown in Figure 5 is from cells isolated from a FACS experiment, while the NS production shown in Figure 1 is from cells that were not manipulated by FACS analysis, a methodology that presents a cellular stress which, in our hands, requires more recovery time *in vitro* following sorting. As a result, the NS cultures in these two distinct experiments were grown at different densities and are not directly comparable. Importantly, E11.5 and P0 cultures were treated and grown in precisely the same manner within each experiment, enabling direct comparison between embryonic and postnatal cultures within that particular experiment.

### Ligand Blocking of Neogenin Receptor Leads to Apoptosis in E11.5 NS

Based on previous work that suggests Neogenin may function as a dependence receptor, as well as by the identification of an active Caspase3 cleavage site in the intracellular region of the protein [25], [26], the effects of ligand-blocking by an anti-Neogenin antibody were determined in E11.5 and P0 NS. There was a greater than 2-fold increase in the number of trypan-blue positive cells in E11.5 NS as compared to P0 NS following incubation with the blocking antibody, using a non-ligand blocking Neogenin antibody as a control (Figure S6). To examine if the cells were undergoing apoptotic death, the levels of activated Caspase3/7 were determined. Following 24h of exposure to 5 µg/ml of a ligand-blocking Neogenin antibody, there were increased levels of activated Caspase3/7 in the E11.5 NS but no significant increases in P0 NS, over the controls (IgG and PBS) (Figure 5F).

### RGMa, a Putative Neogenin Ligand, Is Expressed by Cortical NS

**Figure 6:**
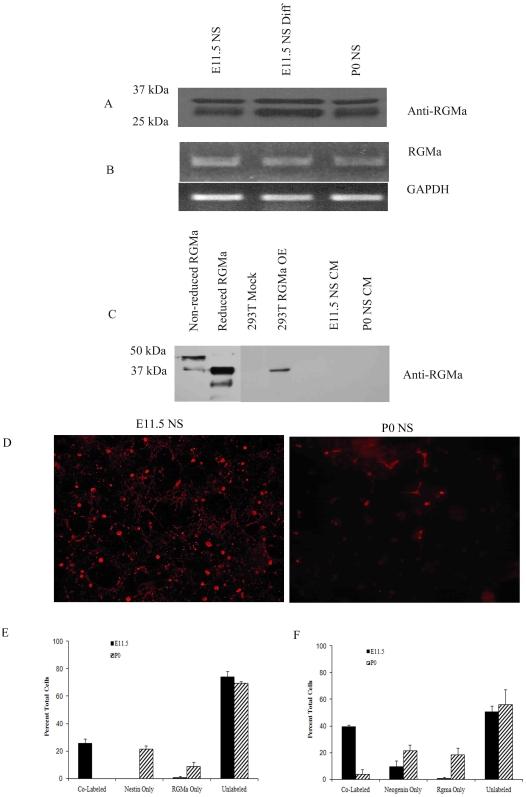
RGMa, a putative Neogenin ligand, is expressed in E11.5 and P0 NS, but is co-expressed with a subset of Nestin-positive and Neogenin-positive cells only in E11.5 NS. RGMa protein expression appeared to be similar across E11.5 and P0 NS and E11.5 differentiated NS (A), as is RGMa mRNA (B). Western blot analysis of concentrated E11.5 and P0 NS conditioned medium (CM) demonstrate NS do not release endogenous RGMa *in vitro* (C). Medium from RGMa-transfected 293T cells, however, contained released RGMa. Presence of RGMa by immunocytochemistry (red, D) illustrates differences in expression patterns *in vitro*. RGMa is co-expressed with the putative stem cell marker, Nestin, in only a subset of cells from E11.5 NS (E). Neogenin and RGMa were co-expressed in E11.5 NS but not in P0, as illustrated in F.

RGMa, a GPI-linked protein, has been previously identified as a high affinity ligand for the Neogenin receptor [27]. To determine if RGMa is expressed in these cultures, message and protein levels were examined by semi-quantitative PCR and western blot analysis. RGMa protein and message were expressed equally in both undifferentiated and differentiated E11.5 and P0 NS (Figure 6A, B). To confirm that RGMa was not being released extracellularly, concentrated cell culture medium from E11.5 and P0 NS was examined by western blot analysis (Figure 6C), with no detectable RGMa in the medium from cells expressing endogenous RGMa. In contrast, exogenous expression of RGMa in 293T cells was identified in the culture medium. *In vitro*, therefore, RGMa appears to be expressed cell autonomously by E11.5 and P0 NS.

By immunocytochemistry, approximately 41% of the dissociated cells in E11.5 NS expressed RGMa while only 22% of the cells from P0 NS expressed RGMa (Figure 6D). In E11.5 NS, RGMa expression appeared to be at the membrane, diffuse and ubiquitous, while in P0 NS, fewer cells expressed RGMa, and the staining appeared cytoplasmic (Figure 6D). In addition, essentially all RGMa positive cells in E11.5 NS expressed Nestin, while there was essentially no co-expression of RGMa and Nestin in P0 NS (Figure 6E). In separate experiments, RGMa and Neogenin were predominantly co-expressed by the same cells in E11.5 (40% of total cells), while only 4% of the P0 cells co-expressed RGMa and Neogenin (Figure 6F).

## Discussion

In these experiments, embryonic (E11.5) and postnatal (P0) murine cortex-derived NS were used as a source of NSPC for the analysis of membrane and membrane-associated proteins. These cultures were characterized to reveal that E11.5 NS contain cells that were highly neurogenic and proliferative as compared to P0 NS. We identified membrane and membrane-associated proteins highly expressed by E11.5 NS, as compared to P0 NS and differentiated E11.5 NS. To demonstrate the relevance of these proteins to stem cell biology, Neogenin, one of the proteins highly expressed by E11.5 NS, was further studied, and while additional experiments are warranted, the protein may play a role in NSPC survival.

### E11.5 NS Contain a Persistently Self-Renewing Stem/Progenitor Cell while P0 NS Are More Restricted

While the NS culture model has its limitations, the use of acutely isolated cortical tissue has its own limitations, specifically that the tissue itself will be a heterogeneous mix of cells (including postmitotic cells) and it is difficult to precisely ascertain the proliferation and potential of this heterogeneous population of cells. The NS culture model attempts to reduce this heterogeneity, as most if not all postmitotic cells are eliminated in the initial passage. We chose the NS culture model, in part because we were able to address the issue of obtaining sufficient starting material, but also because we were able to comprehensively and directly test the proliferative abilities and the potential of the cultures which were being further interrogated by protein expression. The NS-generating cells isolated from E11.5 cortex were able to self-renew, to produce the major cell lineages of the CNS, and to do both with longevity *in vitro*. However, NS-generating cells isolated from the early postnatal cortex demonstrated a reduction in the ability to generate tripotent NS with time, with a subsequent increase in astrogliogenesis over neurogenesis, and a reduced ability to self-renew. As illustrated in Figure 2, the differences that become apparent at D14 P0 cultures are a reflection of the diminished self-renewal and neurogenic capacity of the P0 NSPCs; E11.5 NS in contrast, contain a self-renewing population of multipotent stem-like cells. This is in agreement with previous work demonstrating lineage restriction and reduced self-renewal as cortical development proceeds. Neural stem cells isolated at the peak of neurogenesis (occurring primarily during embryogenesis) and gliogenesis (occurring primarily postnatally) mirror the *in vivo* pattern of cell specification upon differentiation [7]. Cortical progenitor cells derived later in development lack the capacity to generate earlier born cell types, by progression through states of competence, whereby the ability and nature of the response to extrinsic factors, and to generate certain cell types, changes with time [5], [7], [8], [28]. Using an enriched population of cortical neural progenitor cells isolated from E10.5 and E17 and adult cortex, Abramova *et al.* found age specific changes in transcript expression, suggesting that NSPCs with different temporal identities are distinct [10]. Other studies have supported our finding of key differences between neurogenic, more highly self-renewing cells and gliogenic progenitors, regardless of the source. For example, Naka *et al.* utilized expression microarrays to discover that the transcription factors COUP-TF I and II are important regulators the transition from a self-renewing, neurogenic state to a restricted gliogenic state in embryonic stem cell-derived neural progenitors [29]. Although self-renewal is difficult to precisely determine, for our purposes here we refer to the ability of the cells to continue to proliferate *in vitro* and to generate the main cell types of the CNS. As NS are passaged to single-cells, the ability of the culture to meet these criteria hinges on cells being continually produced that are similar (if not identical) to those present in the initial culture with respect to their proliferative abilities and potential. We do acknowledge however, the likelihood that cells evolve in their characteristics over time *in vitro* as they do *in vivo*. Culture effects cannot be ruled out in these experiments, but the fact that E11.5 and P0 NS isolated and grown in precisely the same manner *in vitro* demonstrate significant differences in longevity, potential and protein expression, argues against this being purely a culture artifact. Taken together, the use of E11.5 and P0 NS was a suitable choice to identify proteins associated with NSPC, enabling both the generation of sufficient numbers of cells for the subsequent proteomics experiments while still reflecting biologically distinct and relevant characteristics of NSPC.

### Using Protein Expression Analysis to Interrogate Neural Stem and Progenitor Cells

To further characterize the proliferative and neurogenic cells in the E11.5 NS, highly expressed membrane and membrane-associated proteins specific to this population were identified, over both differentiated E11.5 NS and the more restricted P0 NS. Of those identified, 54 were highly expressed in E11.5 NS over both differentiated E11.5 NS and P0 NS, 24 proteins were comparably expressed in E11.5 and P0 NS over differentiated E11.5 NS, and 13 proteins were highly expressed specifically in P0 NS compared to E11.5 NS. The analysis of hydrophobic membrane proteins continues to present significant challenges to the field of proteomics. No one separation technique has emerged to meet all needs, with both liquid- and gel- based separation techniques presenting specific issues relating to solubility of membrane proteins. In our gel-based approach, we maximized membrane protein representation by utilizing the zwitterionic sulfobetaine detergent, amidosulfobetaine-14 (ASB14), to solubilize the membrane-enriched pellet, a detergent which is also compatible with isoelectric focusing, in addition to overnight isoelectric strip rehydration. In the membrane-enriched fraction, 31% of the identified proteins were classified as membrane or membrane-associated. The remaining identified proteins likely reside in other compartments or in the membranes of other organelles; however, as published sub-proteomics analyses increase, proteins hitherto thought to have predictable sub-cellular locations are increasingly being found in other compartments. Heat shock protein 90 (HSP90) for instance, which has increased expression in E11.5 NS, has been observed in the cytoplasm and the outer membrane, with distinct location-specific functions, and is currently a clinically relevant target in tumour metastasis [30], [31].

Several heterotrimeric G proteins were also highly expressed in E11.5 NS, including guanine nucleotide-binding protein G (o) (alpha subunit 1), guanine nucleotide-binding protein (alpha-12, alpha-13 subunit), and Receptor for Activated C Kinase 1 (RACK1). While the precise function of these proteins in this developmental context is not clear, G proteins are crucial in asymmetric cell division in normal development during neuroblast divisions of *D. melanogaster* [32], and in the initial embryonic divisions of *C. elegans* [33], with similar roles for G protein βγ-subunits in mouse cortical progenitors [34] and human neural progenitors [35], suggesting that further study in the context of neural development is warranted.

The tyrosine kinase receptor, TrkC, was, surprisingly, highly expressed in E11.5 NS. The TrkC receptor, and its preferred ligand neurotrophin-3 (NT-3), has been shown to regulate neuronal differentiation and survival [36], [37], and as such, would be predicted to be expressed to a greater extent in differentiating NS cultures. However, TrkC may be functioning in NSPC survival, as recent work on human ES cells has demonstrated possible roles for NT-3 as a survival factor, mediated through the TrkC receptor and the PI-3K signaling cascade [38], [39].

### Neogenin As a Possible Dependence Receptor during Development

Neogenin was highly expressed in a population of NSPC from E11.5 NS, as compared to the more restricted P0 NS, with overall expression of Neogenin decreasing upon differentiation. To demonstrate the biological relevance of the proteins identified in this study, Neogenin was studied in further detail to examine its role in NSPC biology.

Neogenin has been shown previously to have developmentally distinct functions and has been diversely described as an axonal guidance receptor, as a stabilizer of the mammary gland progenitor cell niche, and in the formation of the neural tube at the earliest points in development [26], [40], [41], [42], [43], [44]. The expression of Neogenin by a proliferative and neurogenic population, as shown in these experiments, is supported by studies in the mouse cortex, whereby Neogenin expression was observed in radial glia, neuroblasts, olfactory neuronal progenitors, and epithelial cells at E12-E14, as well as by Nestin- and GFAP-positive cells within the SVZ surrounding the lateral ventricles in the adult [24], [45]. However, Neogenin is also expressed in cells outside of the proliferative neurogenic niche, as well as in more differentiated cell populations, including Tuj1-positive cells within the intermediate zone of the cortex, newly born (immature) migrating interneurons, PCNA-negative cells in the neuroepithelium at E12.5, and cells within the adult CNS [45]. In this study, Neogenin was predominantly co-expressed with Nestin, a NSPC marker, in E11.5 NS but not in P0 NS. Previous work has shown that enrichment for cells with high levels of Neogenin protein from E14.5 telencephalic lobes resulted in a proliferative and neurogenic culture, as compared to Neogenin-low or negative populations [24]. In the current study, a similar proliferative, neurogenic Neogenin-high population was isolated from E11.5 and adult SVZ NS, but not from the more restricted P0 NS. Thus, within specific set of cells - those derived from E11.5 cortical or adult SVZ NS - Neogenin may serve as a prospective marker to enrich for and study long-term self-renewing neural stem cells. However, Neogenin cannot universally do so, given that in P0 NS the protein is likely expressed by a very different population of cells, and perhaps functioning differently. While we cannot offer the precise identity of the double positive Neogenin/Nestin cell at this point, we have demonstrated that Neogenin is highly expressed by E11.5 NS and that the Neogenin positive cell, isolated from adult SVZ NS and the proliferative, highly neurogenic E11.5 NS is associated with longevity in culture and the ability to continue to produce NS. Neogenin as well as other proteins identified in this study may also serve as markers of NSPC, perhaps most successfully in a combinatorial approach, and will facilitate future studies aimed at understanding subpopulations of NSPC.

The putative GPI-anchored ligand for Neogenin, RGMa, was also expressed by E11.5 and P0 NS cultures. RGMa is a member of a family of Repulsive Guidance proteins, originally isolated as a chemorepulsive molecule [46], [47]. Though RGMa has been described as guidance molecule in the developing retina, the retinal ganglion cell projections are surprisingly normal in RGMa mutant mice; the presence of cephalic neural tube closure defects however, suggests an alternative function for RGMa during development [48].

Despite what is known, the question remains as to the precise role of the Neogenin and RGMa receptor-ligand pair in neural stem cell biology. Previous reports support the development-dependent function of these proteins. At the 2 cell stage in development in *Xenopus*, RGMa1 appears to induce cell death through the Neogenin receptor, while in the developing chick, RGMa modulates the pro-apoptosis activity of Neogenin, to promote neuronal differentiation and cell survival [25], [26], [49]. These functions appear to be replaced with roles in axonal guidance later in development. In light of these reports, the current study raises an important question regarding the developmental function of Neogenin and RGMa. In E11.5 NS, RGMa was expressed in Neogenin- and Nestin-positive cells, while in P0 NS, there was an overall decrease in the expression of these proteins and, importantly, Neogenin and RGMa were expressed in separate cells and no longer associated with co-expression of Nestin. Recent work has led to an emerging theory that Neogenin and RGMa may be functioning as a dependence receptor [26], [50]. In the current experiments, there were no significant consequences of ligand blocking in P0 NS, while application of the same ligand-blocking antibody led to increased activated Caspase3/7 in E11.5 NS.

Taken together, these data suggest that Neogenin and RGMa may have different functions during embryonic and postnatal development in NSPC *in vitro*. While there is limited evidence for this at the transcript level [23], it is intriguing to speculate there may be preferential translation of the Caspase-3-site-deficient isoform of Neogenin later in development [51]. Neogenin and RGMa may promote survival of NSPCs during embryogenesis, perhaps in modulating the number of neural stem and progenitor cells and may assume another role postnatally, perhaps primarily related to axonal pathfinding and guidance. This does stress that the usefulness of Neogenin as a stem/progenitor cell marker is likely to be enhanced when used in a combinatorial manner with other stem cell markers.

In summary, while the embryonic and early postnatal cortex contains a heterogeneous pool of progenitors as reflected in the NS culture model, characterization of these cultures according to their ability to proliferate, to generate the major cell types of the CNS and to do so with longevity, has revealed important differences between NS generated at E11.5 and at P0. These studies have identified membrane and membrane-associated proteins highly expressed by proliferative and neurogenic E11.5 NS, as compared to more restricted P0 NS. The identified proteins are likely candidates for further interrogation, as demonstrated by the studies on the function of Neogenin, and has provided some important insight into the broad protein signature of NSPC. These proteins may also serve as markers of NSPC, perhaps most successfully in a combinatorial approach, as has been recently demonstrated [52], which will facilitate future studies aimed at understanding subpopulations of NSPC.

## Materials and Methods

### Reagents

Tissue culture reagents were obtained from GIBCO-Invitrogen. Basic fibroblast growth factor (bFGF) was obtained from Peprotech. Heparin and protease inhibitor cocktail were obtained from Sigma-Aldrich. The following antibodies were used: anti-O4 (1∶20, Chemicon), anti-Tuj1 (1∶500, Covance), anti-Nestin (1∶50, Rat 401 DSHB-University of Iowa), anti-GFAP (1∶1000, DAKO), anti-HSP90 (1∶1000, Cell Signaling), anti-RACK1 (1∶2500, BD Transduction Laboratories), anti-TrkC (1∶1000, R&D Systems), anti-actin (1∶500, Sigma), anti-Neogenin cytoplasmic (1∶1000, R&D Systems), anti-Neogenin monoclonal extracellular (5 µg/ml, R&D Systems), αRGMa (1∶1000, R&D Systems), and Alexa-conjugated secondary antibodies (1∶2000, Molecular Probes). Propidium iodide (Molecular Probes) was used at 1∶3000. The detergent, ASB14, was purchased from Calbiochem.

### High Density NS Cultures

NS were grown from E11.5 and P0 cortex (including ventricular and subventricular zone, but with minimal ventral cortex) from CD-1 mice (Charles River), as previously described [11], at a density of 50,000 cells/ml medium (DMEM/F12 containing 20 ng/ml bFGF, 5 µg/ml heparin, B27, and penicillin-streptomycin), and passaged every 7 d. Secondary NS were differentiated by removal of bFGF for 72 h. Cells were centrifuged in the presence of protease inhibitors, and stored at -80°C until analyzed.

### Low Density NS Cultures

Low density cultures were analyzed for differentiation potential and longevity. As outlined in Figure S1, Day 1 (D1) low density cultures were derived directly from cortical tissue, while Day 7 (D7) and Day 14 (D14) low density cultures were derived from primary NS, and secondary NS, respectively. Cultures were plated at 1,000 cells/ml medium (Neurobasal medium containing B27, 2 mM L-glutamine, 2 µg/ml heparin, 20 ng/ml bFGF and penicillin/streptomycin) [53], treated as described for high density culture, and the NS were counted following 14 d in culture. The data represent five independent cultures, with each culture counted in triplicate.

### Adult SVZ NS Cultures

SVZ tissue was isolated from adult CD-1 mice, followed by mechanical trituration with TripLExpress (Invitrogen). Cells were grown at 50,000 cells/ml medium (DMEM/F12 containing B27, 20 ng/ml bFGF, 50 ng/ml EGF and penicillin-streptomycin), and passaged every 7 d. Sorting experiments were performed on tertiary SVZ NS.

### Immunocytochemistry

Immunocytochemistry was performed as previously described [11], [54], following 72 h of differentiation using anti-Tuj1, anti-O4, anti-GFAP, and propidium iodide. Individual NS from randomly chosen fields from 4 separate cultures were scored for immunoreactivity and morphology: astrocytes alone (‘A’), neurons and astrocytes (‘N/A’), oligodendrocytes and astrocytes (‘O/A’), and neurons, oligodendrocytes and astrocytes (‘N/O/A’). At least 30 NS and 300 cells were scored per experiment/per condition, with the exception of P0 D14 where only 20 NS and a minimum 100 cells were scored, as a consequence of reduced growth. Data was analyzed by one-way ANOVA, with a p value≤0.05 considered a significant difference.

### Membrane Protein Preparation and Analysis

As outlined in Figure S2, secondary NS were sheared in 1 mM NaHCO_3_/1 mM CaCl_2_ buffer pH 8.0 containing protease inhibitors, and centrifuged for 5 m at 1,000×g (P1), 5 m at 5,000xg (P2) and 1 h at 100,000×g (P3 and Supernatant), with the final protein pellet (P3) solublized in 7 M urea, 2 M thiourea, 2% ASB14, 0.4% Amersham IPG Buffer and 50 mM DTT. Protein concentration was determined using a PlusOne™ 2-D Quant Kit (Amersham Biosciences-GE) and equal amounts of protein were applied (150 µg) to Immobiline DryStrips (18 cm pH 3–10 linear) and were focused using 50 µA per strip at 20°C with the following conditions: 500 V for 1 m, 4 000 V for 1.5 h, 8 000 V for 25 000 Vh (IPGphor Isoelectric Focusing, Amersham Biosciences-GE). Proteins were separated in the second dimension with 10% SDS-PAGE gels (25.5 cm ×20.5 cm ×1 mm, Ettan DALT II, Amersham Biosciences-GE). Gels were stained (SyproRuby, BioRad), visualized and analyzed with PDQuest Gel Imaging software (Molecular Imager FX, BioRad), in automatic match mode with manual editing, with images normalized according to overall signal. Gel analysis was repeated for separate cultures to reach an n = 7 for each of the three groups: E11.5 NS, E11.5 Differentiated NS, and P0 NS. Quantitative comparison of expression used an upper and lower limit factor of 0.5 (2-fold). Statistical comparison of densities was determined by one-way ANOVA. The proteins were excised (Proteome Works Spot Cutter, BioRad), and in-gel digested using sequence grade trypsin (Promega) [55]. The reduced and alkylated peptides were extracted, dried and stored at -80°C. Peptide samples were analyzed by μLC-MS/MS using either an ion trap mass spectrometer (Thermo-Finnigan LCQ-DECA), or a hybrid quadrapole-time-of-flight mass spectrometer (Applied Biosystems QSTAR XL). Tryptic peptides analyzed by ion trap were reconstituted in 10 µl 70% acetic acid and 5 µl injected on to a reverse-phase column (PLRP-S 0.2×150 mm 5 µm 300 Å, Michrom Biosciences) equilibrated in water/acetonitrile/formic acid (95/5/0.1). Peptides were eluted at 3 µl/min over 80 min with an acetonitrile gradient (0 min, 5%; 60 min, 100%; 70 min, 5%). The LCQ-DECA was operated in data-dependent acquisition mode, using a survey scan of 400–1500 m/z, a data-dependent zoom scan, and MS/MS of singly, doubly and triply charged ions. Tryptic peptides analyzed by hybrid mass spectrometry were reconstituted in 0.1% trifluoroacetic acid and separated as described above. The QSTAR XL was operated using an Information Dependent Acquisition (IDA) mode with an ion scan of 375 to 2000 m/z and with MS/MS of ions with a charge state of 2–5. LCQ-DECA MS/MS data sets were analyzed by Sonar MS/MS software (Genomic Solutions, Version 2004.01.15.01), and QSTAR MS/MS data sets were analyzed by Mascot software (Matrix Science), with reference to databases from NCBI, SIB and EBI. An Expect score of greater than 1×10^−2^ was considered a positive identification. Search parameters included +/− 2 Da precursor, +/− 0.4 Da fragment, 3 missed cleavages by trypsin, carboxyamidomethylation of cysteines, and oxidized methionines.

### Western Blot Analysis

Protein expression was confirmed by western blot analysis using total cell lysate or the P3 fraction. Protein concentration was determined using the Bradford assay (BioRad) and equal amounts of protein were loaded. To determine presence of released RGMa, medium was collected from each culture (24–48 h post-transfection for exogenously produced RGMa), centrifuged, concentrated, and 30 µl of each was analyzed by Western blot.

### Semi-Quantitative PCR

Semi-quantitative RT-PCR was used to evaluate mRNA levels, with GAPDH as a control. The following primer sets were used to examine the expression of various transcripts: Neogenin sense ggg tca aga atg ggg atg tgg tta, antisense ctc tcc tgg ctg gct ggt att ctc; RGMa sense tct tcg acc tcc tca cga ct, antisense atg gtg cca agg aga atc tg.

### Fluorescent Activated Cell Sorting

Flow cytometry was performed in the UCLA Jonsson Comprehensive Cancer Center Flow Cytometry Facility using the Becton Dickinson FACSVantage SE and FACSAriaII High-Speed Cell Sorter Flow Cytometers. E11.5 and P0 NS were grown at 5,000 to 10,000 cells/ml, while adult SVZ NS were grown at 200,000 cells/ml. Cells were exposed to 10 µg/ml anti-Neogenin antibody (extracellular, non-ligand blocking, gift of Dr. H. Cooper) for 1 h followed by exposure to Alexa 488 secondary antibody for 1 h. Sorted E11.5 and P0 cells were grown at 5,000 cells/ml in complete Neurobasal medium for two passages, followed by passages at low density (1,000 cells/ml). NS were counted in each culture prior to passaging. Adult SVZ NS were sorted into a 96-well plate containing medium at 20 cells per well and allowed to grow for 2 weeks before assessing sphere formation. Both antibodies to the extracellular portion of Neogenin labeled the same cells *in vitro* (data not shown).

### Neogenin Ligand-Blocking Antibody and Apoptosis Assay

E11.5 and P0 NS were grown at 2,500 to 10,000 cells/ml. Anti-Neogenin antibody was added at 2.5 to 10 µg/ml of medium and cells were grown for 24 h. Hydrogen peroxide, anti-mouse IgG, and PBS were used as positive and negative controls for the apoptosis assay. Caspase3/7 activities were measured using the Caspase-Glo 3/7 Assay (Promega), according to the manufacturer's protocol, and luminescence was detected using the Analyst HT Microplate Reader (LJL Biosystems).

## Supporting Information

Table S1 Partial list of proteins with higher expression in both E11.5 and P0 NS as compared to differentiated E11.5 NS.Proteins were identified by μLC-MS/MS. Detailed information regarding protein identification can be found in Table S2.
(0.03 MB DOC)

Table S2 List of all proteins identified by μLC-MS/MS. One of two programs was used to establish protein identification: Mascot or Sonar. The respective scores for each protein are listed, as well as the identified peptide sequence. LCQ-DECA MS/MS data sets were analyzed by Sonar MS/MS software (Genomic Solutions, Version 2004.01.15.01), and QSTAR MS/MS data sets were analyzed by Mascot software (Matrix Science), with reference to databases from NCBI, SIB and EBI. An Expect score of greater than 1×10-2 was considered a positive identification. Search parameters included +/− 2 Da precursor, +/− 0.4 Da fragment, 3 missed cleavages by trypsin, carboxyamidomethylation of cysteines, and oxidized methionines.
(0.24 MB PDF)

Figure S1 Neurosphere culture design. Acutely dissociated telencephalon (E11.5) or cortex (P0) from CD1 mice was grown at both high density (50,000 cells/ml) and low density (1,000 cells/ml, designated D1) as described. Subsequent D7, D14, and D21 low density cultures were derived from fully mature high density 1°, 2°, or 3° NS, respectively. High density cultures were passaged every 7d, and low density cultures every 14d.
(0.34 MB TIF)

Figure S2 Proteomics workflow for identification of differentially expressed proteins in NS by 2DGE. Additional details regarding the methodology can be found in the Materials and Methods section.
(4.00 MB TIF)

Figure S3 Representative total ion chromatograph and MS/MS spectrum. A total ion chromatograph (inset, B) and MS/MS spectrum (A) of a tryptic peptide of guanine nucleotide-binding protein beta subunit 2-like 1, a heterotrimeric G protein. The MS/MS spectrum shown is focused in the mass range where the strongest b and y ions are present.
(2.32 MB TIF)

Figure S4 Protein expression for several identified proteins. Western blot analysis of the membrane-enriched preparation for TrkC, Rack1, and HSP90 of E11.5 NS (lane 1), E11.5 Differentiated NS (lane 2), P0 NS (lane 3). The loading control, Actin, was used for total cell lysate. Protein loading was equal in all lanes and measured by Bradford analysis.
(0.10 MB TIF)

Figure S5 Neogenin is highly expressed in E11.5 NS and is co-expressed with Nestin in a sub-population of cells. By immunocytochemistry (A), Neogenin (green) and Nestin (blue) expression was evident in both E11.5 and P0 NS, although there was higher expression of both in E11.5 NS (top panel, A). The percent of cells expressing these proteins is shown in (B). Cell nuclei are shown in red (propidium iodide).
(0.09 MB TIF)

Figure S6 Incubation with ligand-blocking anti-Neogenin antibody increases percent of tryphan-blue positive cells in E11.5 cells. E11.5 and P0 cells were incubated with either the ligand-blocking anti-Neogenin antibody or the cell sorting anti-Neogenin antibody for 3h and tryphan blue positive cells were counted.
(0.06 MB TIF)
