# Supplementary material for: Identification of Differentially Expressed Proteins in Murine Embryonic and Postnatal Cortical Neural Progenitors
Source: PLoS One. 2010 Feb 9;5(2):e9121. doi: 10.1371/journal.pone.0009121 (PMC2817745; doi:10.1371/journal.pone.0009121)
Supplement: Table S1 — Partial list of proteins with higher expression in both E11.5 and P0 NS as compared to differentiated E11.5 NS.Proteins were identified by μLC-MS/MS. Detailed information regarding protein identification can be found in Table S2. (0.03 MB DOC) [file pone.0009121.s001.doc]

| **Protein Name** | **Gene Symbol** | **UniProtKB/TrEMBL Number** |
| --- | --- | --- |
| Veph-A, Veph-B protein | Veph1 | Q8K4P6 |
| B-cell stimulating factor-3 | Bsf3 | Q9QZM3 |
| Atp5b protein | Atp5b | Q8CI65 |
| Spermatogenesis associated factor | Spata5 | Q9CXZ7 |
| Guanine nucleotide-binding protein | Gnb2l1 | P68040 |
| Protein disulfide-isomerase A4 | Pdia4 | P08003 |
|  |  |  |
