# Supplementary material for: Identification of Differentially Expressed Proteins in Murine Embryonic and Postnatal Cortical Neural Progenitors
Source: PLoS One. 2010 Feb 9;5(2):e9121. doi: 10.1371/journal.pone.0009121 (PMC2817745; doi:10.1371/journal.pone.0009121)
Supplement: Table S2 — List of all proteins identified by μLC-MS/MS. One of two programs was used to establish protein identification: Mascot or Sonar. The respective scores for each protein are listed, as well as the identified peptide sequence. LCQ-DECA MS/MS data sets were analyzed by Sonar MS/MS software (Genomic Solutions, Version 2004.01.15.01), and QSTAR MS/MS data sets were analyzed by Mascot software (Matrix Science), with reference to databases from NCBI, SIB and EBI. An Expect score of greater than 1×10^-2^ was considered a positive identification. Search parameters included +/− 2 Da precursor, +/− 0.4 Da fragment, 3 missed cleavages by trypsin, carboxyamidomethylation of cysteines, and oxidized methionines. (0.24 MB PDF) [file pone.0009121.s002.pdf]

Proteins Highly Expressed in E11.5 NS Compared to P0 NS and E11.5 Diff NS

Protein Name	Gene Symbol	UniProtKB/TrEMBL Accession Number	Genebank Accession Number	Number of Peptides	Sonar Expect Value	Mascot Mowse Score	Peptide Sequence
Voltage-dependent anion channel 2	Vdac2	Q99L98	BC003731	4		107	LTLSALVDGK
							GFGFGLVK
							LTFDTTFSPNTGK
							MCIPPSYADLGK
Neogenin	Neo1	P97798	Y09535	2		42	AAEREAGR
							DVVASLVSTR
Sodium-calcium exchanger	Slc8a1	O35157	AF004666	4	2.8x10-2		IWNETVSNLTLMALGSSAPEILLSVIEVCGHNFTAGDLGPSR
							VGIIDDDIFEEDENFLVHLSNVR
							IFFEQGTYQCLENCGTVALTIMRR
							LVALVALLFSHVDHITADTEAETGGNETTECTGSYYCK
Neurotrophic tyrosine kinase receptor	TrkC	Q6VNS1	AY336094	2	2.9x10-2		AGHSVIGMTR
							MDVSLCPAK
Seizure related gene 6	Sez6	Q7TSK2	BC053011	2	6.0x10-2		AVAKAPAASSALDAAHLAAAIFLPLVAMVLLVGGVYR
							THPRPYNR
Adenylate cyclase, type IX	Adcy9	P51830	U30602	5		46	TFDNLK
							CFPQTQRR
							DLEVEKALKER
							FDSMNLEEACLER
							AIEQFCQEKKEMVNMR
TGF-beta receptor type III	Tgfbr3	O88393	BC070428	2	2.2×10-2		DSFQASGYPGLELTLLDPTCKAK
							GTMGRPQEVHVLNLR
Guanine nucleotide-binding protein G(o), alpha subunit 1	Gnao1	P18872	M36777	2		77	LLLLGAGESGK
							TTGIVETHFTFK
Guanine nucleotide-binding protein, alpha-12, alpha-13 subunit	Gna12/Gna13	P27600	M63659	1		65	ILLLGAGESGK
GTP-binding protein REM 1	Rem1	O35929	U91601	1		50	VVLLGDPGVGK
Guanine nucleotide binding protein, beta 2, related sequence 1	Gnb2-rs1	Q5NCC6	AL645849	2		97	DKTIIMWK
							DVLSVAFSSDNR
Guanine nucleotide-binding protein beta subunit-like protein (RACK1)	Gnb2l1	Q9CSQ0	AK012242	3		93	IIVDELK
							DVLSVAFSSDNR
							IIVDELKQEVISTSSK
Similar to interleukin 17 receptor E isoform 1	Il17re	Q6AZ51	BC078742	2	3.6x10-2		TRPVLLLHAADSEAQR
							IGPLPWLWAAR
Hypothetical protein C6B12.02c in chromosome I	SPAC6B12.02c	O14207	Z98531	2		43	FDGAR
							AAAKSAIAR
Glycoprotein endo-alpha-1,2-mannosidase-like protein	Gm50	Q6P1J0	AAH65047	2	6.0x10-2		MPLNIISDSVYVVNAVLALETAGNFKQSSPVSEILR
							LQYVHVSIDTSSGVLHASPLTGEK
Receptor tryosine-protein kinase erbB-2	Erbb2	Q6ZPE0	AK129487	2		30	ACPPCAPTCK
							GRLPTDCCHEQCAAGCTGPK
Alpha 3 catenin - Mus musculus adult male testis cDNA, RIKEN full-length enriched library	Ctnna3	Q8C0N3	AK030166	1		21	KIHPVQVMSEFRGRQVY
FK506 binding protein 9	Fkbp9	Q80ZZ6	BC043129	1	1.3×10-2		IITVPPFLAYGEEGDGK
Coatomer epsilon subunit	Cope	O89079	NP_067513	1	9.0 x 10-2		TLHQGDGLECMAMTIQILLK
Mus musculus 10 days neonate olfactory brain cDNA, RIKEN full-length enriched library	Glud1	Q8C273	AK089152	4	1.0×10-6		DSNYHLLMSVQESLER
							ELEDFKLQHGSILGFPK
							IIAEGANGPTTPEADKIFLER
							LGEVLLLSR
DNA-binding protein Ikaros	Znfn1a1	Q03267	L03547	4	5.5x10-13		SNHSAQDAVDNLLLLSK
							SGLIYLTNHINPHAR
							MLDASGEKMNGSHR
							SHTERPFQCNQCGASFTQK
Enoyl-CoA hydratase, mitochondrial	Echs1	Q8BH95	AK040391	2	1.7x10-4		AQFGQPEILLGTIPGAGGTQR
							LFYSTFATDDRR
Aldolase 1, A isoform	Aldoa	Q6NY00	BC066801	3	2.1×10-6		YTPSGQSGAAASESLFISNHAY
							IGEHTPSALAIMENANVLAR
							MPHPYPALTPEQK
Mus musculus ES cells cDNA, RIKEN full-length enriched library	2410129E14Rik/Tubb2	Q9CWF2/Q7TMM9	AK010786/BC055441	6	3.1×10-11		GHYTEGAELVDSVLDVVR
							LHFFMPGFAPLTSR
							GHYTEGAELVDSVLDVVRK
							ALTVPELTQQMFDSK
							ALTVPELTQQMFDSKNMMAACDPR
							EIVHIQAGQCGNQIGAK
Brix domain containing protein 1	Bxdc1	Q9JJ80	BC025093	1		47	QDLSKLQTRKMK
Sulfotransferase 1C1	Sult1c1	Q9D939	AY005469	2	9.8x10-3		SILDQSISSFMR
							VVWGSWFDHVK
78 kDa glucose-regulated protein (Hsp70)	Hspa5 (Grp78)	P20029	M19351	4	6.4 x10-4		SFYPEEVSSMVLTK
							TTPSYVAFTDTER
							IINEPTAAAIAYGLDKK
							NQVAMNPTNTVFDAK
Chaperonin containing TCP-1 theta subunit	Cct8	Q9WVS5	BAA81879	8	5.6x10-17		TAEELMNFSKGEENLMDAQVK
							FAEAFEAIPR
							IGLSVSEVISGYEIACK
							AIAGTGANVIVTGGK
							LATNAAVTVLR
							DMLEASILDTYLGK
							DVDEVSSLLR
							IAVYSCPFDGMITETK
T-complex protein 1, alpha subunit B	Cct1	P11983	M12899	3	2.7x10-3		SQNVMAAASIANIVK
							EQLAIAEFAR

							YINENLIINTDELGR
T-complex protein 1, beta subunit	Cct2	P80314	Z31553	8	7.0x10-9		DAALMVTNDGATILK
							QVLLSAAEAAEVILR
							GATQQILDEAER
							LSSFIGAIAIGDLVK
							LALVTGGEIASTFDHPELVK
							VQDDEVGDGTTSVTVLAAELLR
							ILKHGINCFINR
							AETAR
T-complex protein 1, epsilon subunit	Cct5	P80316	Z31555	5	1.8x10-10		LGFAGVVQEISFGTTK
							DVDFELIKVEGK
							GSNDMQYQHVIETLIGK
							AVTIFIR
							LMVELSK
26S protease regulatory subunit 4	Psmc1	P62192	NP_032973.1	6	7x10-7		ENAPAIIFIDEIDAIATK
							EAVELPLTHFELYK
							MNLSEEVDLEDYVARPDK
							LIFSTITSK
							IQDEIPALSVSRPQTGLSFLGPEPEDLEDLYSR
							ILLELLNQMDGFDQNVNVK
26S protease regulatory subunit 6B	Psmc4	P54775	AB040869	4	3.5x10-5		LQQELEFLEVQEEYIKDEQK
							ENAPAIIFIDEIDAIATK
							IQDEIPALSVSRPQTGLSFLGPEPEDLEDLYSR
							MNLSEEVDLEDYVARPDK
26S protease regulatory subunit 6A	Psmc3	O88685	AB040858	6	2.0x10-10		GATELTHEDYMEGILEVQAK
							APSIIFIDELDAIGTK
							QTYFLPVIGLVDAEK
							EKAPSIIFIDELDAIGTK
							LAGPQLVQMFIGDGAK
							VDILDPALLR
Proteasome subunit beta type 6	Psmb6	Q60692	MMU13393	3	1.6x10-10		TTIMAVQFDGGVVLGADSR
							SGSAADTQAVADAVTYQLGFHSIELNEPPLVHTAASLFK
							QVLLGDQIPK
Proteasome subunit alpha type 5	Psma5	Q9Z2U1	AF019661	3	1.1x10-4		LFQVEYAIEAIK
							AIGSASEGAQSSLQEVYHK
							ITSPLMEPSSIEK
Proteasome beta 4 subunit	Psmb4	Q91VV7	NP_032971	3	2.3x10-2		QVLGQMVIDEELLGDGHSYSPR
							TQNPMVTGTSVLGVK
							VNNSTMLGASGDYADFQYLK
Heterogeneous nuclear ribonucleoproteins A2/B1	Hnrpa2b1	O88569	AF073993	3	2.4x10-10		LFIGGLSFETTEESLR
							IDTIEIITDR
							GGGGNFGPGPGSNFR
Heterogeneous nuclear ribonucleoprotein F	Hnrpf	Q9Z2X1	BC018185	1	4.1x10-2		ITGEAFVQFASQELAEK
Heterogeneous nuclear ribonucleoprotein K (Hnrpk protein)	Hnrpk	Q5FWJ5	BC089328	11		486	DLAGSIIGK
							GGDLMAYDR
							VVLIGGKPDR
							GSDFDCELR
							NTDEMVELR
							NLPLPPPPPPR
							IDEPLEGSEDR
							SRNTDEMVELR
							LFQECCPHSTDR
							RPAAEDMEEEQAFKR
							TDYNASVSVPDSSGPER
Ribosomal protein S2	Rps2	Q9DC49	AK004568	1		57	GCTATLGNFAK
Elongation factor 1-delta	Eef1d	P57776	BAB30841.1 or AAG17466	1	8.9x10-3		GVVQDLQQAISK
Elongation factor 1-beta	Eef1b	O70251	BC039635	1	9.4x10-4		TPAGLQVLNDYLADK
Trip12 protein	Trip12	Q99KN5	NP_598736	2	3.0x10-2		QETSLTSHDLFDIDPVVARSVYHLEDIVR
							LFLQFVTGSPR
Thioredoxin-like protein 1	Txnl1	Q8CDN6	AF052660	5	1.7x10-4		IDQYQGADAVGLEEK
							GYMDLMPFINK
							IFINLPR
							SEPTQALELTEDDIKEDGIVPLR
							MVGVKPVGSDPDFQPELSGAGSR
Creatine kinase, B chain	Ckb	Q04447	M74149	9	4.2x10-19		LAVEALSSLDGDLSGR
							FCTGLTQIETLFK
							LEQGQAIDDLMPAQK
							LGFSEVELVQMVVDGVK
							SMTEAEQQQLIDDHFLFDKPVSPLLLASGMAR
							GTGGVDTAAVGGVFDVSNADR
							VLTPELYAELR
							TDLNPDNLQGGDDLDPNYVLSSR
							MPFSNSHNTQK
MKIAA4061 protein	B230396K10Rik	Q5DTQ5	AK220465	2		44	QELLKANGEIK
							QELLKANGEIKHVSSLLAKMEK
Eukaryotic translation initiation factor 3, subunit 12	Eif3s12	Q58EU9	BC091749	4		44	ANVGK
							ANVGKLLK
							CMIDQAHQEERPIR
							YNPENLATLER
Eukaryotic initiation factor 4A- I and II	Eif4a1	P60843	MMEIF4AI	1		61	KGVAINMVTEEDKR

Calreticulin	Calr	P14211	BC003453	4		133	FYALSAK<br>LFPSGLDQK<br>VHVIFNYK<br>FYGDLEKDK
Actin, cytoplasmic 2	Actg1	P63260	X13055	8		202	GILTLK<br>AVFPSIVGR<br>GYSFTTTAER<br>EITALAPSTMK<br>QEYDESGPSIVHR<br>SYELPDGQVITIGNER<br>VAPEEHPVLLTEAPLNPK<br>TTGIVMDSGDGVTHTVPITEGYALPHAILR
Tropomyosin 3, gamma	Tpm3	Q8K0Z5	BC029186	3		84	TIDDLEDKLK<br>IQLVEEELDR<br>ALKDEEKMELQEIQLK
F-actin capping protein alpha-2 subunit	Capza2	P47754	U16741	3		35	LLLNNDNLLR<br>KVDGQQTIIACIESHQFQAK<br>IVEAAENEYQTAISENYQTMSDTTFK
Pyruvate kinase, isozyme M2	Pkm2	P52480	D38379	2		84	GDLGIEIPAEK<br>NTGIICTIGPASR
Heat shock protein HSP 90-beta	Hspcb	P11499	M36829	6	4.0×10-9		GVVDSEDLPLNISR<br>ADLINNLGTIAK<br>TLTLVDTGIGMTK<br>IDIIPNPQER<br>ALLFIPR<br>RAPFDLFENK

Proteins Highly Expressed in P0 NS Compared to E11.5 NS and E11.5 Diff NS

Protein Name	Gene Symbol	UniProtKB/TrEMBL Accession Number	Genebank Accession Number	Number of Peptides	Sonar Expect Value	Mascot Mowse Score	
Cyclic nucleotide gated channel alpha 2	Cnga2	Q80XH6	BC048775	2		33	DGEGKGTKKK<br>GPELQTVTTHQGDGK
Selectin P	SELP	Q5TI45	AL022146	2		32	WSATPPTCK<br>IPERGNMTCLHSAK
ATPase, H+ transporting, V0 subunit D isoform 1	Atp6v0d1	Q921S5	BC011075	2		108	LYPEGLAQLAR<br>LLFEGAGSNPGDK
Arsenical pump-driving ATPase	Asna1	O54984	AF039405	5		296	LIQELAK<br>LEETLPVIR<br>LPLLPHEVR<br>LLNFPTIVER<br>TTCSCSLAVQLSK
Mothers against decapentaplegic homolog 9 (smad8/smad9)	Smad9	Q9JIW5	AF175408	2		42	ALSCPGQPSK<br>KGAMDELERALSCPGQPSK
Down-regulated by Ctnnb1, a	Drctnnb1a	Q6P9N1	BC060692	2		32	CIQVEITPTSSR<br>EGTRCIQVEITPTSSR
Mus musculus adult male medulla oblongata cDNA, RIKEN full-length enriched library	Tra1	Q8CCY5	AK031918	4		133	SGYLLPDTK<br>IYFMAGSSR<br>EAESSPFVER<br>EGVKFDESEKTK
Damage-specific DNA binding protein 1	Ddb1	Q9WV39	AF159853	3		77	LYDGLFK<br>LFMLLLEK<br>QSGESIDIITR
Adaptor protein complex AP-1, beta 1 subunit	Ap1b1	Q5SVG4	AL645522	11		593	NINLIVQK<br>KGEIFELK<br>LLSTDPVAAK<br>GLEISGTFTR<br>LQSSNIFTVAK<br>AELNSDKKEKK<br>DCPLNTEAASNK<br>MEPLNNLQVAVK<br>IQPGNPSFTLSLK<br>QAGSISMDLQLTNK<br>NVEGQDMLYQSLK
Mus musculus 2 days neonate thymus cDNA, RIKEN full-length enriched library	5330429B09Rik	Q8C2C1	AK088897	1		22	LGTWLPR
Chromodomain helicase-DNA-binding protein 4 (CHD-4)	Chd4	Q8BM83	AK034549	2		25	AYHMVCLDPDMEK<br>AYHMVCLDPDMEKAPEGK
N-ethylmaleimide sensitive fusion protein	Nsf	Q923C6	BC006627	2		38	GFSDIFR<br>HVRGILLYGPPGCGK
Early endosome antigen 1	Eea1	Q8BL66	AK046231	2		31	QTTVIQDLK<br>GKAAVLDLEK

Proteins Highly Expressed in Both E11.5 NS and P0 NS Compared to E11.5 Diff NS

Protein Name	Gene Symbol	UniProtKB/TrEMBL Accession Number	Genebank Accession Number	Number of Peptides	Sonar Expect Value	Mascot Mowse Score	
Veph-A, Veph-B protein	Veph1	Q8K4P6	AB085187	1		35	RDRSLPR
(Il-6 superfamily) B-cell stimulating factor-3	Bsf3	Q9QZM3	AF176913	1		26	LGAETLPR
Atp5b protein	Atp5b	Q8CI65	BC037127	12	3.7×10-28		TVLIMELINNVAK

							IMDPNIVGNEHYDVAR
							VLDSGAPIKIPVGPETLGR
							TIAMDGTEGLVR
							LDATTVLSR
							VVDLLAPYAK
							FLSQPFQVAEVFTGHMGK
							SLQDIIAILGMDELSEEDKLTVSR
							IMNVIGEPIDER
							LVPLKETIK
							GLAGTQPFGGSATGAR
							IPSAVGYQPTLATDMGTMQER
Spermatogenesis associated factor	Spata5	Q9CXZ7	AK011111	2	4.0x10-2		GLLLYGPPGTGK
							QEVYTAWPVAGFPGGK
Guanine nucleotide-binding protein beta subunit 2-like 1	Gnb2l1	P68040	BC046760	3	9.0x10-3		IIVDELKQEVISTSSK
							DETNYGIPQR
							TIIMWK
Dynactin subunit 2	Dctn2	Q99KJ8	BC004613	1	1.3×10-3		LLGPDAAINLADPDGALAK
Annexin A5	Anxa5	P48036	U29396	3		166	TPEELSAIK
							VLTEIIASR
							GTVTDFPGFDGR
Glyceraldehyde-3-phosphate dehydrogenase	Gapd	P16858	M32599	2	2.3x10-2		VIHDNFGIVEGLMAVHAITATGQK
							VIISAPSADAPMFVMGVNHEK
Alpha enolase	Eno1	P17182	AAH03891.1	2		72	AAVPSGASTGIYEALELR
							LAMQEFMILPVGASSFR
Catalase	Cat	P24270	M62897	1		66	FYTEDGNWDLVGNNTPIFFIR
Malate dehydrogenase 2, NAD	Mdh2	Q8R1P0	BC023482	4	1.4×10-6		VDFPQDQLATLTGR
							VAVLGASGGIGQPLSLLLK
							TIIPLISQCTPK
							IFGVTTLDIVR
Transitional endoplasmic reticulum ATPase	Vcp	Q01853	Z14044	7	7.8×10-17		NAPAIIFIDELDAIAPK
							IVSQLLTLMDGLK
							LDQLIYIPLPDEK
							NVFIIGATNRPDIIDPAILRPGR
							MTNGFSGADLTEICQR
							GVLFYGPPGCGK
							MDELQLFR
Tumor rejection antigen gp96	Tra1	Q91V38	BC011439	7	9.3x10-14		GVVDSDDLPLNVSR
							TDDEVVQREEEAIQLDGLNASQIR
							ESDDPMAYIHFTAEGEVTFK
							FAFQAEVNR
							ELISNASDALDKIR
							DDEVDVDGTVEEDLGK
							EEASDYLELDTIK
Heat shock cognate 71 kDa protein	Hspa8	P63017	U27129	5		153	DAGTIAGLNVLR
							TTPSYVAFTDTER
							IINEPTAAAIAYGLDK
							NQVAMNPTNTVFDAK
							IINEPTAAAIAYGLDKK
Acidic ribosomal phosphoprotein P0	Arbp	Q5FWB6	BC089496	5	4.1x10-13		TSFFQALGITTK
							VLALSVETEYTFPLAEK
							AFLADPSAFAAAAPAAAATTAAPAAAAAPAK
							IIQLLDDYPK
							GTIEILSDVQLIK
60S acidic ribosomal protein P2	Rplp2	P99027	BC012413	2	2.4x10-5		NIEDVIAQGVGK
							YVASYLLAALGGNSSPSAK
40S ribosomal protein SA	Rpsa	P14206	NP_035159.2	7	1.6x10-17		AIVAIENPADVSVISSR
							FTPGTFTNQIQAAFR
							FAAATGATPIAGR
							SDGIYIINLK
							KSDGIYIINLK
							LLVVTDPR
							FTPGTFTNQIQAAFREPR
Lamr1 protein	Lamr1	Q58E74	BC092041	4	1x10-13		FLAAGTHLGGTNLDFQMEQYIYK
							AIVAIENPADVSVISSR
							SDGIYIINLK
							KSDGIYIINLK
Protein disulfide-isomerase A4	Pdia4	P08003	AAA39907.1	5	1.4x10-11		MHVMDVQGSTEASAIKDYVVK
							VEGFPTIYFAPSGDK
							RFDVSGYPTLK
							DLGLSESGEDVNAAILDESGKK
							GRPFDYNGPR
Protein disulfide-isomerase	P4hb	P09103	X06453	5	9.8x10-11		VDATEESDLAQQYGVR
							QFLLAAEAIDDIPFGITSNSGVFSK
							VLVGANFEEVAFDEKK
							YQLDKDGVVLFK
							HNQLPLVIEFTEQTAPK
Protein disulfide-isomerase A6	Pdia6	Q922R8	AK076558	3	5.8x10-16		GSTAPVGGGSFPTITPR
							LAAVDATVNQVLASR
							TGEAIVDAALSALR
Protein disulfide-isomerase A3	Pdia3	P27773	M73329	1	2.5×10-3		SDVLELTDENFESR
Vimentin	Vim	P20152	M24849	2		52	FLEQQNK
							FADLSEAANR
