# Supplementary figures and images for: Identification of Differentially Expressed Proteins in Murine Embryonic and Postnatal Cortical Neural Progenitors
Source: PLoS One. 2010 Feb 9;5(2):e9121. doi: 10.1371/journal.pone.0009121 (PMC2817745; doi:10.1371/journal.pone.0009121)

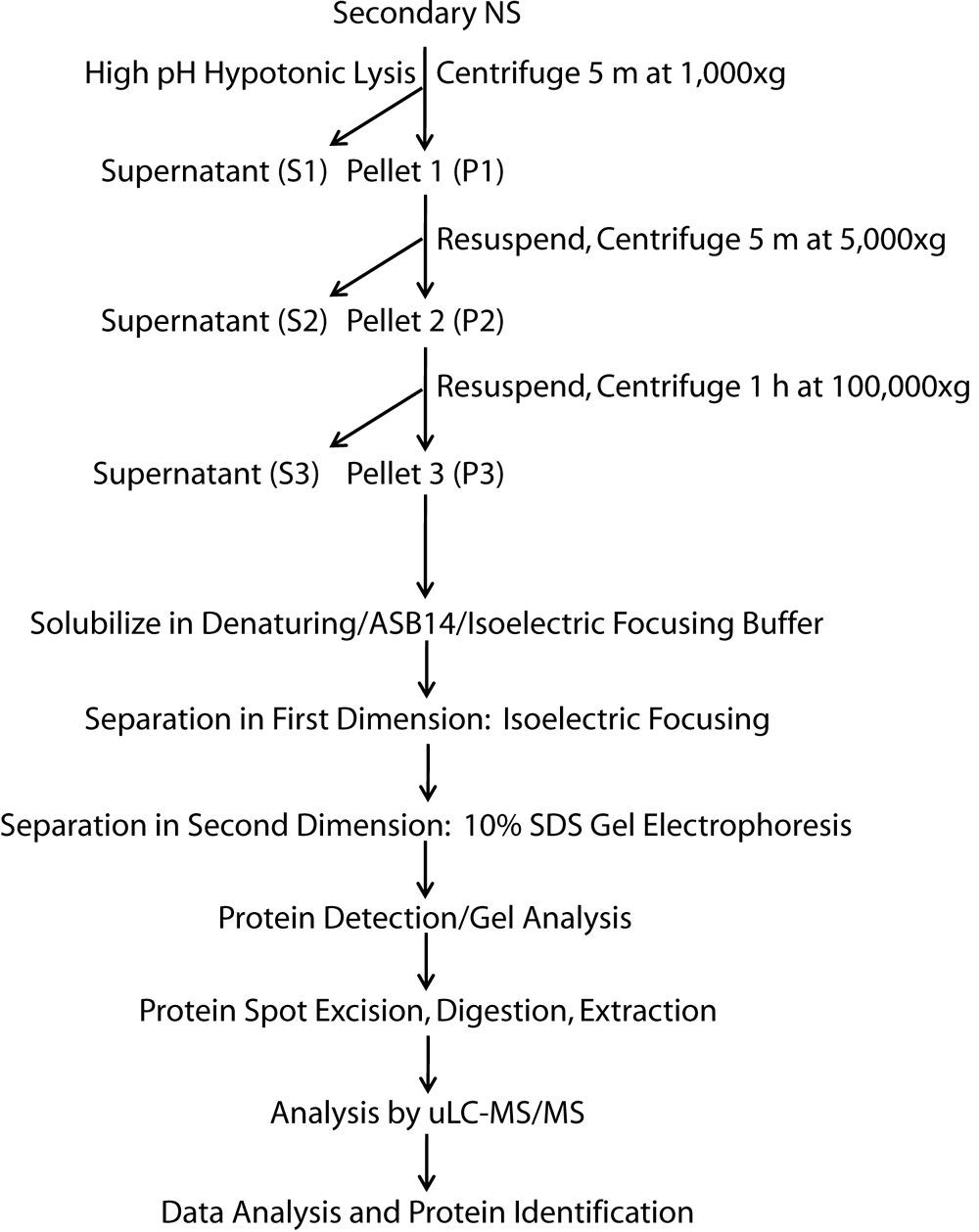

Supplement: Figure S2 — Proteomics workflow for identification of differentially expressed proteins in NS by 2DGE. Additional details regarding the methodology can be found in the Materials and Methods section. (4.00 MB TIF) [file pone.0009121.s004.tif]

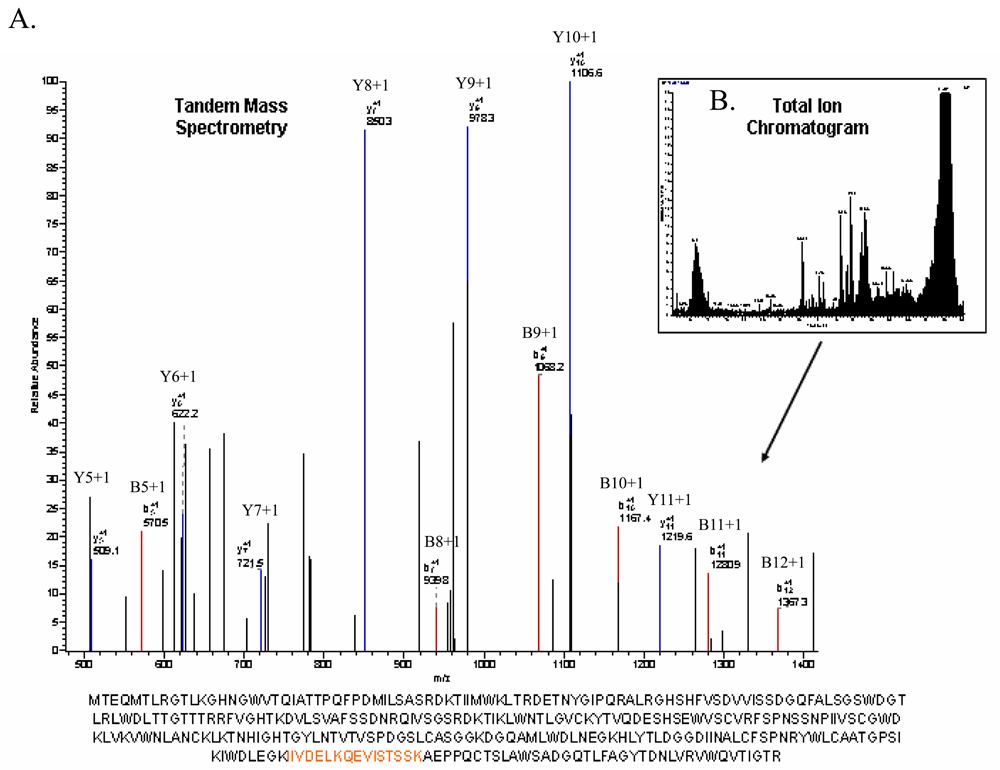

Supplement: Figure S3 — Representative total ion chromatograph and MS/MS spectrum. A total ion chromatograph (inset, B) and MS/MS spectrum (A) of a tryptic peptide of guanine nucleotide-binding protein beta subunit 2-like 1, a heterotrimeric G protein. The MS/MS spectrum shown is focused in the mass range where the strongest b and y ions are present. (2.32 MB TIF) [file pone.0009121.s005.tif]

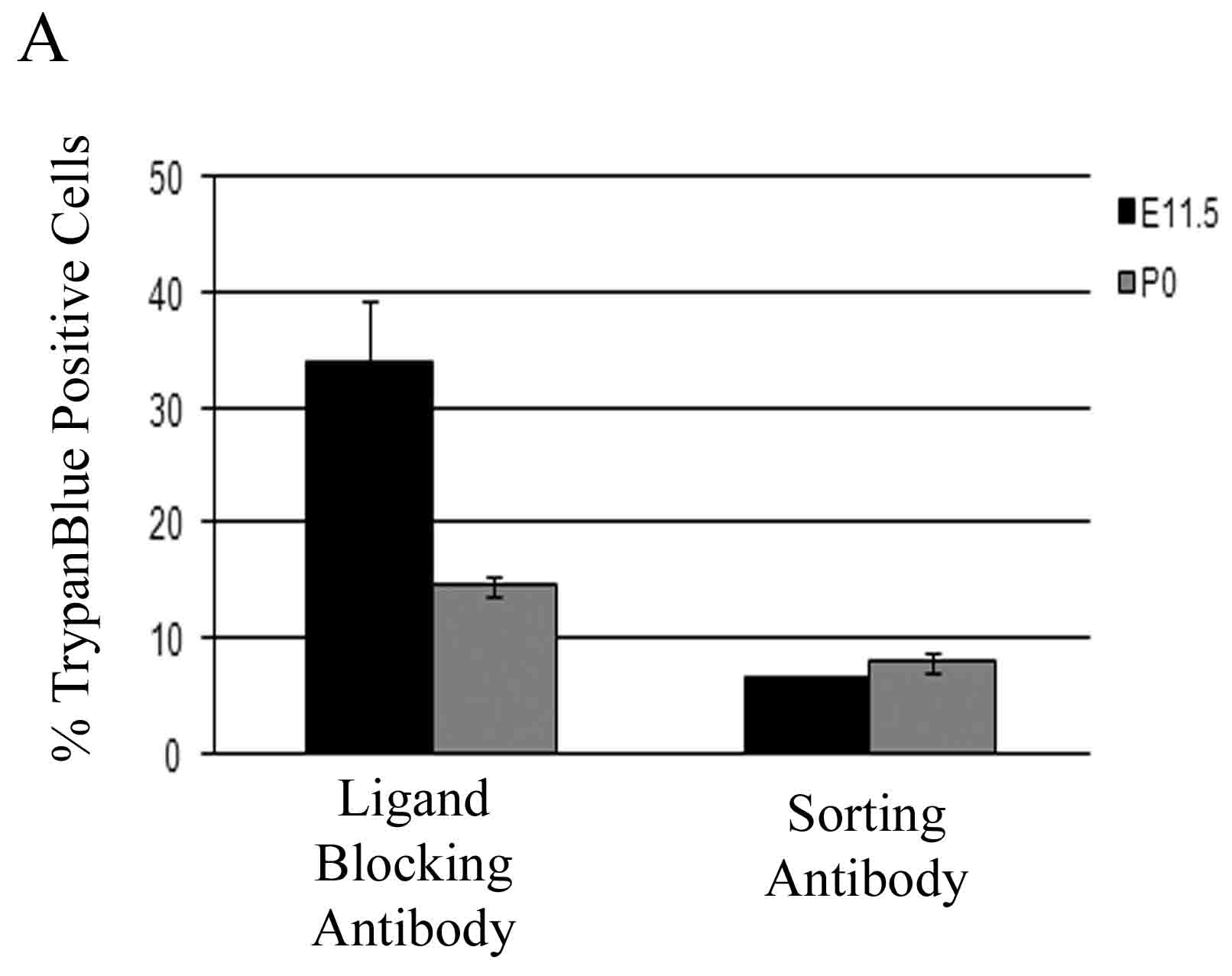

Supplement: Figure S6 — Incubation with ligand-blocking anti-Neogenin antibody increases percent of tryphan-blue positive cells in E11.5 cells. E11.5 and P0 cells were incubated with either the ligand-blocking anti-Neogenin antibody or the cell sorting anti-Neogenin antibody for 3h and tryphan blue positive cells were counted. (0.06 MB TIF) [file pone.0009121.s008.tif]
